# Towards a Novel Patch Material for Cardiac Applications: Tissue-Specific Extracellular Matrix Introduces Essential Key Features to Decellularized Amniotic Membrane

**DOI:** 10.3390/ijms19041032

**Published:** 2018-03-29

**Authors:** Matthias Becker, Janita A. Maring, Maria Schneider, Aarón X. Herrera Martin, Martina Seifert, Oliver Klein, Thorsten Braun, Volkmar Falk, Christof Stamm

**Affiliations:** 1 Charité—Universitätsmedizin Berlin, Corporate Member of Freie Universität Berlin, Humboldt-Universität zu Berlin, and Berlin Institute of Health, 13353 Berlin, Germany; matthias.becker@charite.de (M.B.); josemaring@hotmail.com (J.A.M.); maria.schneider@charite.de (M.Sc.); aaron.herrera@charite.de (A.X.H.M.) martina.seifert@charite.de (M.Se.); oliver.klein@charite.de (O.K.); 2 Berlin-Brandenburg Center for Regenerative Therapies (BCRT), 13353 Berlin, Germany; falk@dhzb.de; 3 Julius Wolff Institute for Biomechanics and Musculoskeletal Regeneration, 13353 Berlin, Germany; 4 Department of Obstetrics and Gynecology, Charite Medical University, 13353 Berlin, Germany; thorsten.braun@charite.de; 5 German Centre for Cardiovascular Research (DZHK), Partner Site Berlin, 13316 Berlin, Germany; 6 Deutsches Herzzentrum Berlin (DHZB), Augustenburger Platz 1, 13353 Berlin, Germany

**Keywords:** extracellular matrix, hydrogel, cardioprotection, patch, epicardium, amnion, immunocompatibility

## Abstract

There is a growing need for scaffold material with tissue-specific bioactivity for use in regenerative medicine, tissue engineering, and for surgical repair of structural defects. We developed a novel composite biomaterial by processing human cardiac extracellular matrix (ECM) into a hydrogel and combining it with cell-free amniotic membrane via a dry-coating procedure. Cardiac biocompatibility and immunogenicity were tested in vitro using human cardiac fibroblasts, epicardial progenitor cells, murine HL-1 cells, and human immune cells derived from buffy coat. Processing of the ECM preserved important matrix proteins as demonstrated by mass spectrometry. ECM coating did not alter the mechanical characteristics of decellularized amniotic membrane but did cause a clear increase in adhesion capacity, cell proliferation and viability. Activated monocytes secreted less pro-inflammatory cytokines, and both macrophage polarization towards the pro-inflammatory M1 type and T cell proliferation were prevented. We conclude that the incorporation of human cardiac ECM hydrogel shifts and enhances the bioactivity of decellularized amniotic membrane, facilitating its use in future cardiac applications.

## 1. Introduction

Cardiovascular disease remains the leading cause of death in industrialized societies and is on the rise in developing countries [1]. For end-stage heart failure, there is currently no effective therapeutic alternative other than heart transplantation or mechanical assist device implantation, because the regenerative potential of the postnatal mammalian myocardium is insufficient to restore function to the damaged heart [2]. Intramyocardial or intravascular cell transplantation techniques were considered promising regenerative strategies but largely underperformed in clinical trials. For instance, Hastings et al. [3] compared intramyocardial injections of different stem and progenitor cell products and demonstrated the limited performance in in vivo models and clinical applications. This lack of efficacy can be explained by the poor retention of injected cells in the infarcted tissue [4], and it has been published that the application of therapeutics via an epicardial patch system is more effective in creating a sustained effect [5].

As a translationally competent biologic human scaffold system, we have previously shown that decellularized human amniotic membrane alone reduces post-infarct remodeling processes if applied epicardially [6]. However, its pro-regenerative and immunomodulatory capacity may be further enhanced by modulating the protein composition and incorporating target tissue-specific bioactivity [7,8]. So far, the majority of compounds used for these types of studies (e.g., Gelatin, collagen) have been highly defined prior to their use and were investigated individually [9,10,11], and we hypothesized that the use of an organ-specific protein composition would benefit the integration of the patch and aid regenerative processes.

We have previously succeeded in isolating Extracellular Matrix (ECM) from human myocardium (hcECM) with a more preserved protein composition compared to protocols reported by other groups [12], for example, with regards to glycosaminoglycans (GAG) [13], and demonstrated its capacity to support cell viability and induce cardiac lineage differentiation of pluripotent stem cells [14]. Furthermore, we demonstrated that the additional processing to microparticles and subsequent reconstitution as a hydrogel does not affect the cytoprotective capacity of the hcECM [15].

Here, we designed a novel composite patch material, linking the essential biological characteristics of target organ ECM with a stable carrier material by combining hcECM hydrogel (hgECM) with the cell-free amniotic membrane and tested its cytoprotective and immunological properties.

## 2. Results

### 2.1. Processing into Hydrogel Preserves Protein Composition

Processing into a hydrogel via Pepsin digestion may have a significant impact on ECM protein composition, greatly depending on the individual digestion and processing procedure [16,17]. We therefore examined the final protein composition of the hgECM after processing using mass spectrometry (MS).

**Figure 1:**
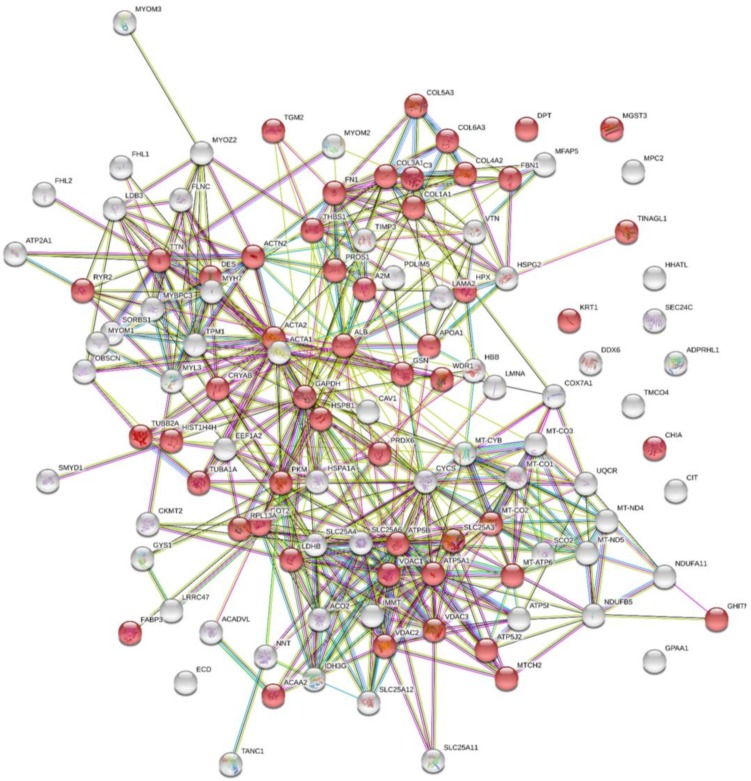
STRING analysis depicting hgECM proteins identified by MS. A total of 113 proteins were identified in the hgECM, of which 51 belong to the ECM region (red spheres). Lines indicate known protein interaction from curated databases (turquoise) and experimentally determined (pink), predicted interactions for gene neighborhood (green), gene fusion (red) and gene co-occurrence (blue). Other connections indicate text mining (yellow), co-expression (black) and protein homology (light blue). Detailed information on identified proteins is provided in Table S1.

A total of 113 proteins were identified in hgECM, of which 51 belong to the Extracellular Region Part (Figure 1) according to the STRING network database (Table S1), indicating that processing of the myocardium by decellularization, pulverization and homogenization preserved the majority of typical ECM proteins. For instance, different subtypes of collagen types 1, 3, 4, 5 and 6 were detected, as well as dermatopontin, fibrillin 1 and fibronectin 1, which are responsible for cell adhesions and Transforming Growth Factor beta (TGF-β) regulation. Other proteins involved in cell adhesion and matrix interaction were found including thrombospondin 1—a cell-to-cell and cell-to-matrix mediator—and vitronectin—a cell-to-substrate adhesion molecule and inhibitor of the membrane-damaging effect of the terminal cytolytic complement pathway.

Furthermore, several intracellular proteins were found that are involved in cell cycle, ECM regulation, or signaling pathway modifications such as acetyl-CoA acyltransferase 2, actin, actinin, different ATP synthases, complement component 3, desmin, chitinase, and heat shock 27 kDa protein 1. Thus, processing not only preserved a large number of ECM proteins but also cellular proteins relevant for cytoprotective support.

### 2.2. DeAM Coating with hgECM Generates Novel Surfaces

Cell-free hcECM slices were processed into microparticles and homogenized into a hydrogel [13] to be used as a coating for decellularized amniotic membrane (DeAM). Scanning electron microscopy (SEM) was performed to visualize the shape of particles and the modified surface of hgECM coated DeAM (DeAM + E) in contrast to DeAM.

**Figure 2:**
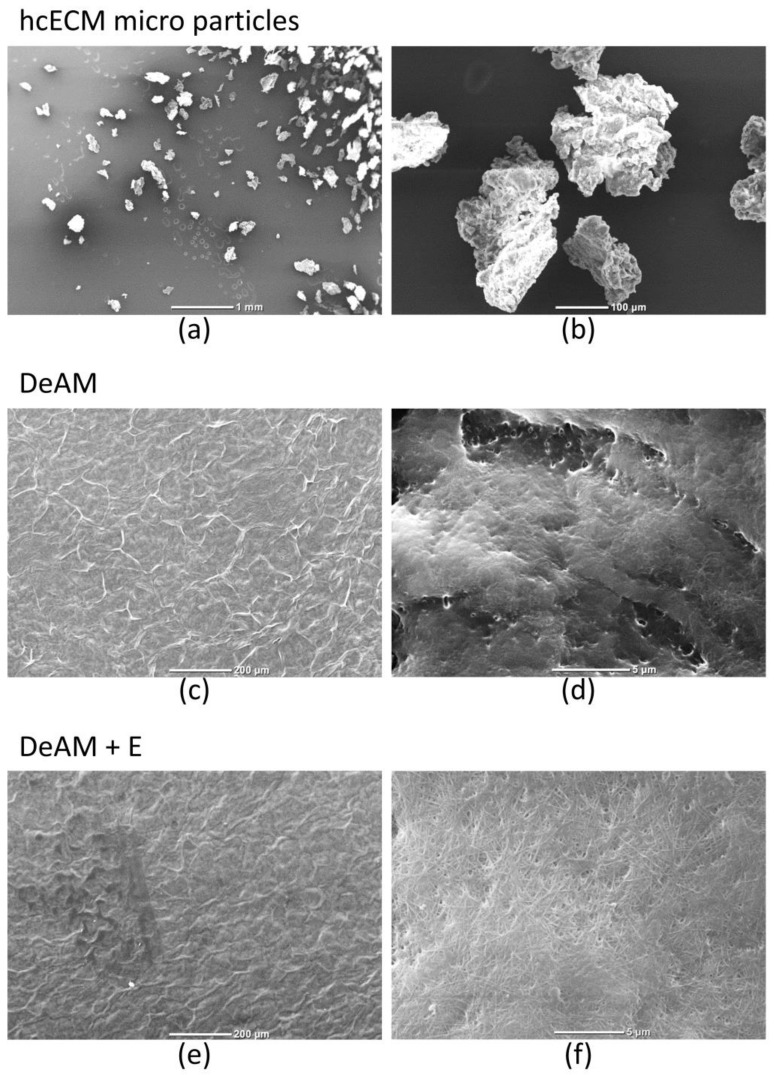
Scanning electron microscope visualization of hcECM microparticles as well as hgECM coated and uncoated DeAM. Structure analysis of patch components is shown as representative images. (**a**,**b**) SEM visualization of hcECM microparticles with a median particle feret diameter of 66 µm [15] ((**a**): magnification 20×; scale bar 1 mm; (**b**): magnification 170×; scale bar 100 µm); (**c**,**d**) Structure of DeAM ((**c**): magnification 100×; scale bar 200 µm; (**d**): magnification 5000×; scale bar 5 µm); (**e**,**f**) DeAM + E ((**e**): magnification 100×; scale bar 200 µm; (**f**): magnification 4500×; scale bar 5 µm).

Pulverization of the hcECM resulted in microparticles with essentially uniform size and shape (Figure 2a,b). Further processing yielded a viscous hydrogel that was used to coat the DeAM carrier material. The structure of uncoated DeAM is shown in (Figure 2c,d). Coating of DeAM with hgECM (DeAM + E) did not change the global morphology as seen with low magnification (Figure 2e), but higher magnification revealed the hgECM generated an additional, unique nano-scale structure on top of the DeAM fibrillar architecture (Figure 2f) providing a modified structural surface.

### 2.3. Manipulation and Processing Do Not Affect the Mechanical Properties of Amniotic Membrane Scaffold Material

The elastic modulus (E-modulus) is an important factor of a material that determines how a material elastically deforms when pressure is applied, such as the pressure placed on the walls of the heart by its movement. Once applied to the organ, the material must withstand the present stress, e.g., up to 0.5 × 10^6^ Pa in the human heart [18]. Uniaxial pulling tests were performed for the different membranes to obtain the E-moduli as the relationship between the stress and strain of the linear region of the load curve. Maximum stress was obtained as the maximum load before sample rupture. However, rupture of the membranes occurred at the border near the clamps due to the stress concentrators produced by the patterned surface of the clamps, leading to micro-fractures in these areas that later propagate and lead to failure of the material. Therefore, the maximum stress values shown here represent a conservative case of the maximum stress that these membranes can withstand.

**Figure 3:**
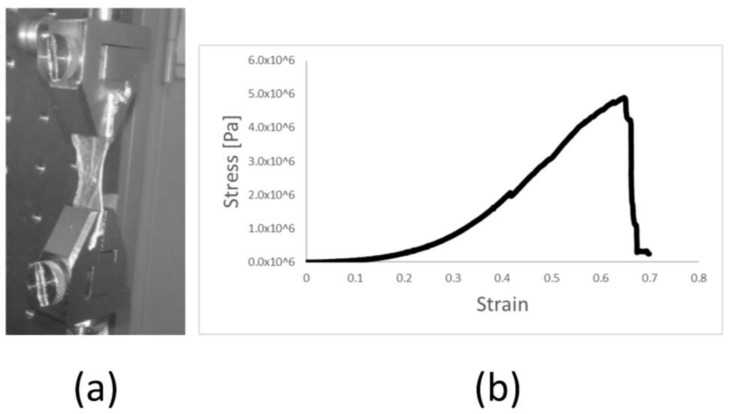

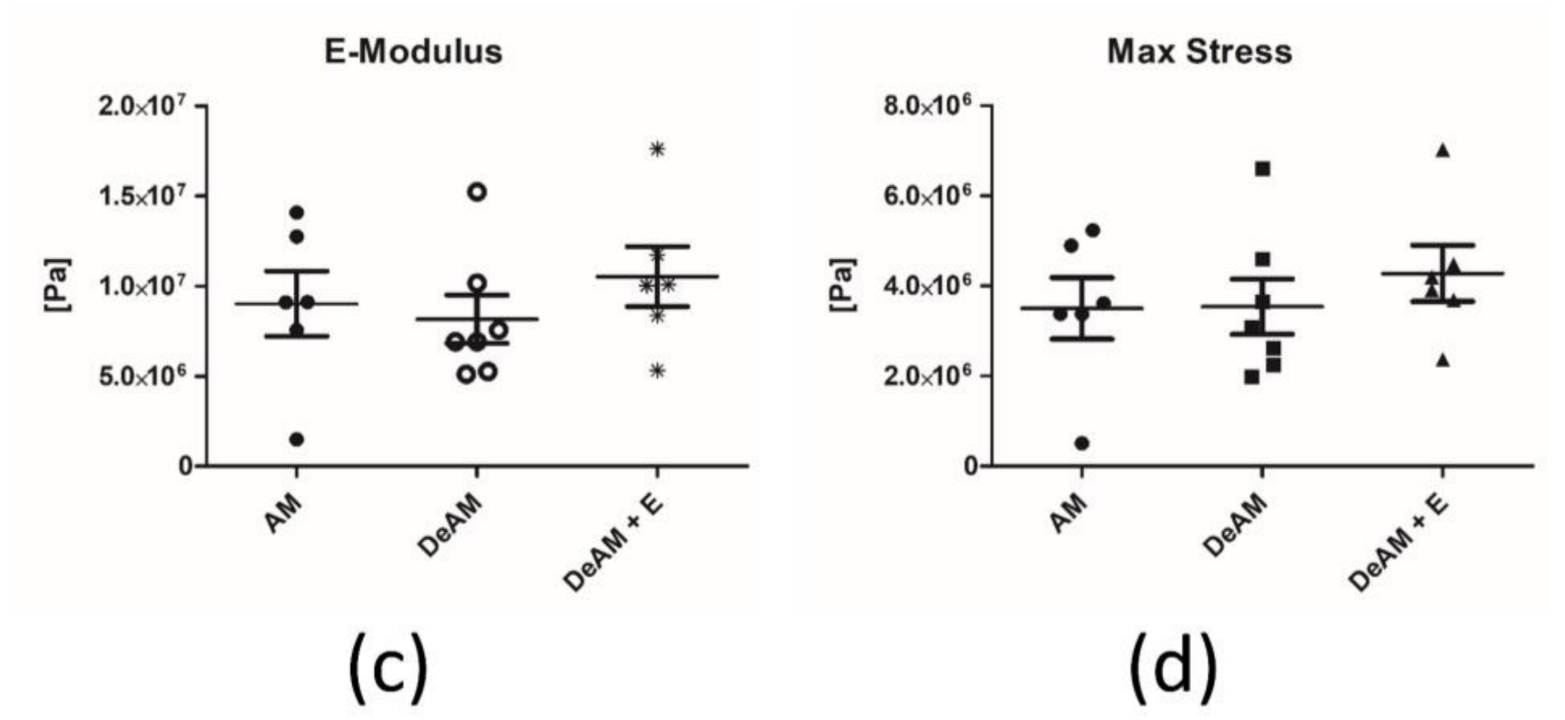
Determination of mechanical properties of the amniotic membrane (AM), the DeAM and the DeAM + E by the uniaxial pulling test. (**a**) Setup of horizontal pulling test, (**b**) Representative stress-strain curve, (**c**) single values E-modulus and (**d**) single values maximum stress resistance. *n* ≥ 6.

We examined the mechanical properties (E-modulus and maximum stress resistance) of the amniotic membrane (AM), the DeAM and the DeAM + E, to elucidate whether the processing of the AM affects the elastic behavior and stress resistance. Typical mechanical behavior of the membranes during a uniaxial pulling test was characterized by a toe region where the load was transferred to sample, a linear region where the E-modulus was obtained, and rupture once the maximum stress has been reached (Figure 3a). Graphical representation of the stress shows the limit of stress retention by the rapid decrease in Pa, while the slope indicates the E-modulus (Figure 3b). The mechanical properties for the analyzed membranes were not significantly different. The average E-moduli (Figure 3c) of AM, DeAM and DeAM + E were 8.8 × 10^6^ ± 1.7 × 10^6^ Pa, 8.2 × 10^6^ ± 1.5 × 10^6^ Pa and 9.9 × 10^6^ ± 1.7 × 10^6^ Pa, respectively. Maximum stress (Figure 3d) for the different membranes was 3.5 × 10^6^ ± 1.7 × 10^6^ Pa, 3.5 × 10^6^ ± 1.6 × 10^6^ Pa and 4.3 × 10^6^ ± 1.5 × 10^6^ Pa for AM, DeAM and DeAM + E respectively. These data confirm that the modified amniotic membrane has mechanical characteristics similar to those of native amniotic tissue. The maximum stress of the membranes is above the stress that the cardiac tissue is subjected to in vivo, indicating that membranes will withstand the physiological loads without failure. Therefore, they are suitable for implantation, surgical application and ultimately in clinical interventions.

### 2.4. hgECM Coating of DeAM Improves Cell Adhesion and Increases Viability

**Figure 4:**
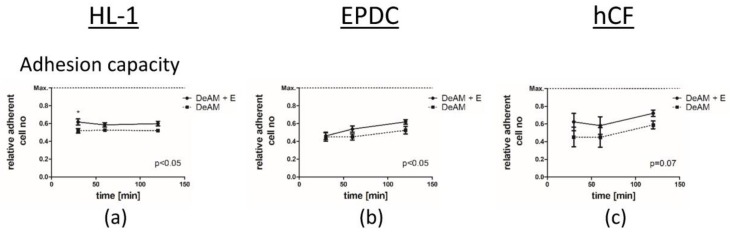

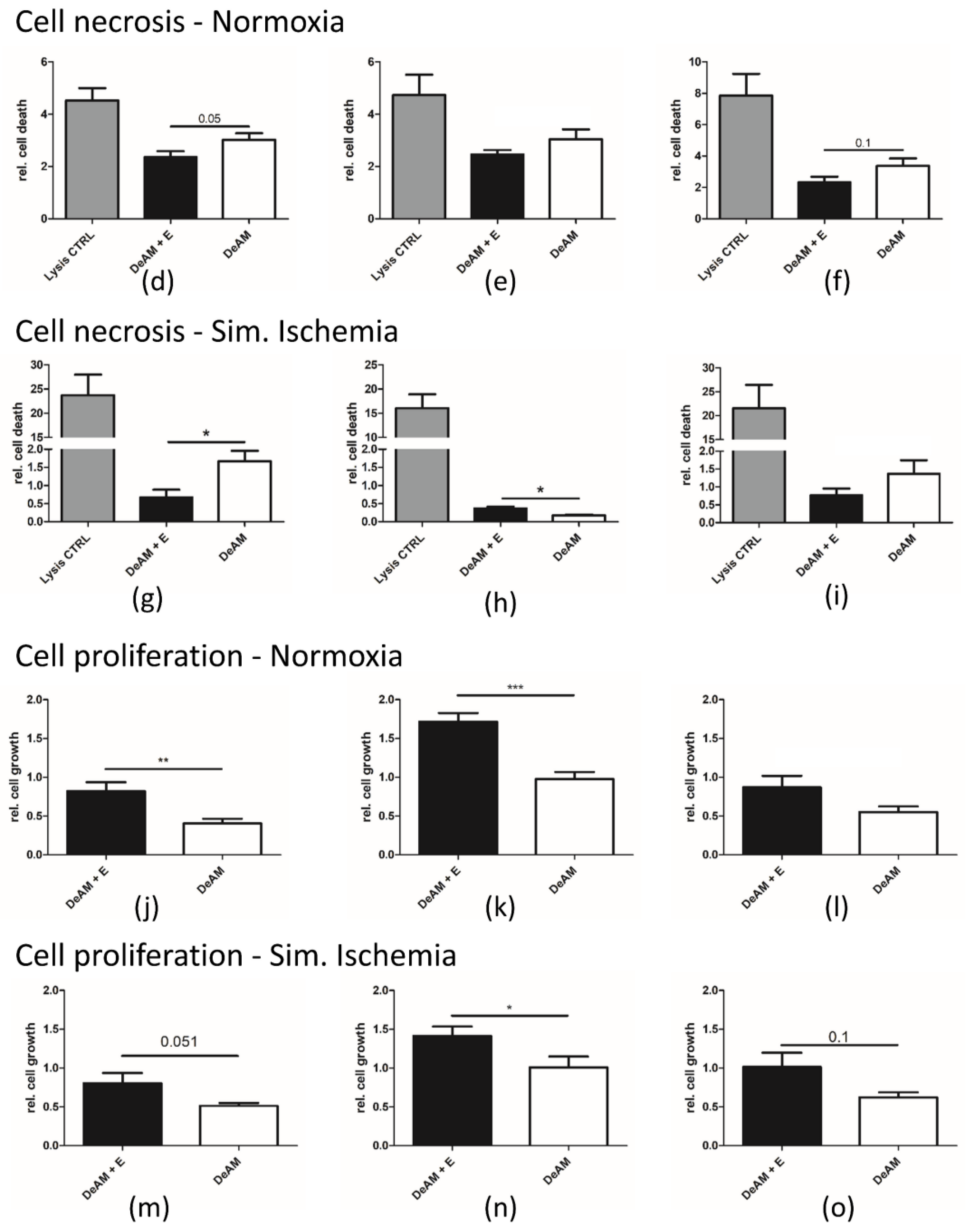
Interaction and viability of HL-1 cells, EPDCs and hCF cultured on DeAM and DeAM + E scaffolds. Adhesion capacity of contractile (**a**) HL-1 cells, (**b**) EPDCs and (**c**) hCF was determined via calcein staining on DeAM (dotted line) and DeAM + E (solid line). Cell necrosis was determined by measuring LDH release of HL-1 cells, EPDCs and hCF under normoxia (**d**–**f**) and “simulated ischemia” (**g**–**i**) cultured on DeAM + E (black) and DeAM (white). Lysis control (grey) indicates total cell death. Cell growth was determined by measuring BrdU-incorporation of HL-1 cells, EPDCs and hCF under normoxia (**j**–**l**) and “simulated ischemia” (**m**–**o**) cultured on DeAM + E (black) and DeAM (white). * *p* < 0.05, ** *p* < 0.01, *** *p* < 0.001; *n* ≥ 3.

To examine the influence of hgECM coating on the biological properties of DeAM, we focused on selected cell types that are relevant for cardiac regeneration processes, namely, human cardiac fibroblasts (hCF), human epicardial derived cells (EPDC), and murine cardiomyocyte-like cells (HL-1), both under normal culture conditions and in “simulated ischemia” (1% O_2_, serum and glucose deprivation) (Figure 4).

On DeAM + E, HL-1 cells adhered better within the first 30 min, but no further increase in adhesion was observed during longer cultivation periods (Figure 4a). In contrast, EPDCs (Figure 4b) showed improved adhesion capacity on DeAM + E only after 120 min. Human cardiac fibroblasts (hCF) (Figure 4c) displayed the trend towards increased adhesion on DeAM + E (*p* = 0.07).

Culturing on DeAM + E reduced cell death for all tested cell types under normoxic conditions (Figure 4d–f). Under “simulated ischemia” conditions, necrosis of HL-1 cells substantially decreased when cultured on DeAM + E (Figure 4g). In the case of EPDCs exposed to “simulated ischemia”, this effect was reversed, despite remarkable minimization of LDH-release on both surfaces compared to standard cell culture conditions (normalized to value 1). In addition, hCF also benefited from culturing on DeAM + E under “simulated ischemia” conditions and displayed less cell necrosis (Figure 4i).

The cell growth rate was improved on DeAM + E for all cell types as seen by BrdU incorporation in HL-1 cells, EPDCs, and hCF, both under normoxia and in “simulated ischemia” (Figure 4j–o), highlighting the dominant advantage of the modified scaffold provided by DeAM + E.

### 2.5. Coating with hgECM Modulates Inflammatory Responses

#### 2.5.1. Pro-inflammatory Cytokine Release

**Figure 5:**
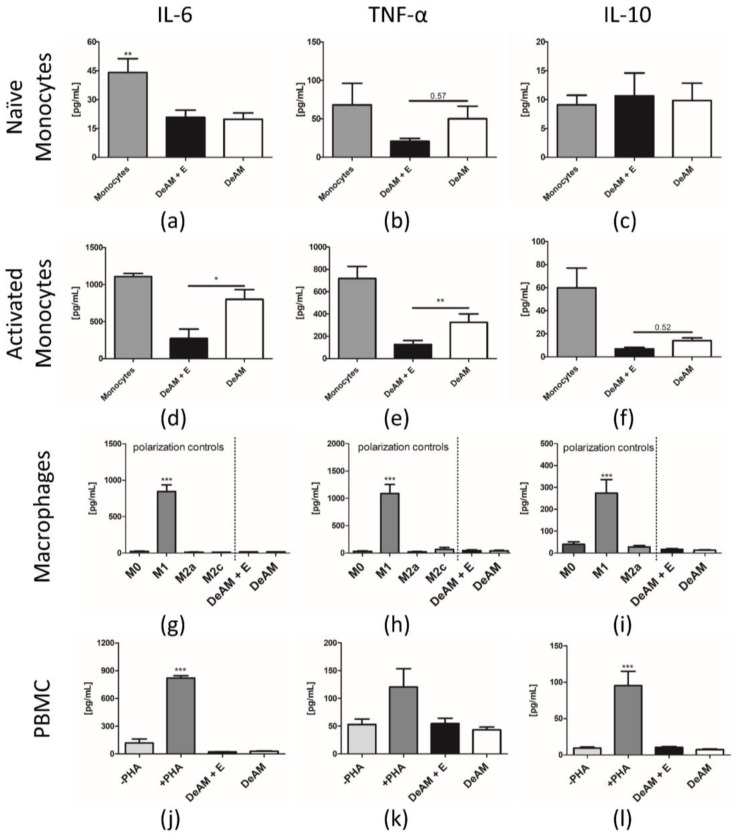
Cytokine release from monocytes, macrophages and PBMCs cultured on DeAM and DeAM + E scaffolds determined by ELISA. Supernatants were collected after 24 h of naïve ((**a**–**c**) ** *p* < 0.01 to all groups) and LPS-activated ((**d**–**f**) * *p* < 0.05, ** *p* < 0.01) CD14+ monocytes on DeAM + E (black), DeAM (white) or monocyte standard culture control conditions (grey). Macrophages derived from CD14^+^-monocytes (M0) were polarized towards pro-inflammatory M1- and anti-inflammatory M2a- and M2c-type. After 2 days, supernatants were collected and analyzed for IL-6, TNF-α and IL-10 ((**g**–**i**) *** *p* < 0.001 to all groups) concentration. PBMCs from human buffy coat were cultured for 5 days. Supernatants were collected and analyzed for cytokine secretion of IL-6, TNF-α and IL-10 ((**j**–**l**) *** *p* < 0.001 to all groups). The positive control group was stimulated with PHA. *n* ≥ 3.

Inflammation plays an exceedingly important role in myocardial remodeling as well as in spontaneous and induced regeneration processes. Any biologic implant must be able to balance pro- and anti-inflammatory stimuli to exert appropriate and sustained functional effects. We therefore studied the effect of DeAM, hgECM coated DeAM on the pro- and anti-inflammatory cytokines IL-6, TNF-α, and IL-10 secreted from human peripheral blood mononuclear cells (PBMCs), and monocytes as well as CD14^+^-derived macrophage subpopulations (Figure 5).

Compared to standard culture plate conditions, cytokine secretion of naïve monocytes was affected by DeAM both with and without hgECM coating. IL-6 secretion was significantly decreased on both surfaces (Figure 5a) but the release of TNF-α (Figure 5b) was reduced only in monocytes cultured on DeAM + E. The secretion of IL-10 was unchanged on either surface (Figure 5c). Lipopolysaccharide (LPS)-activated monocytes demonstrated a marked suppression in the secretion of IL-6 (Figure 5d) and TNF-α (Figure 5e) on DeAM + E. However, no difference in IL-10 secretion was observed in the presence of DeAM + E or DeAM (Figure 5f).

Macrophages derived from M-CSF stimulated CD14^+^ monocytes once polarized towards the pro-inflammatory M1 type were unequivocally identifiable by a substantial increase in all measured cytokines IL-6, TNF-α and IL-10 (Figure 5g–i) compared to all control groups as well as macrophages cultured on DeAM and DeAM + E. Thus, polarization towards pro-inflammatory M1 type on DeAM as well as DeAM with additional hgECM coating did not occur. Because IL-10 stimulation was used to induce M2c polarization, IL-10 secretion by the M2c control could not be determined (Figure 5i).

The cytokine secretion profile of PBMCs demonstrated clearly that only the Phytohemagglutinin (PHA) stimulated PBMC (positive control) secreted significantly more IL-6, TNF-α and IL-10 (Figure 5j–l) than all other groups including PBMCs cultured on both DeAM and DeAM + E. Therefore, the cytokine secretion profiles of cultured PBMCs were not stimulated by DeAM or DeAM + E.

#### 2.5.2. Macrophage Polarization and T Cell Proliferation

**Figure 6:**
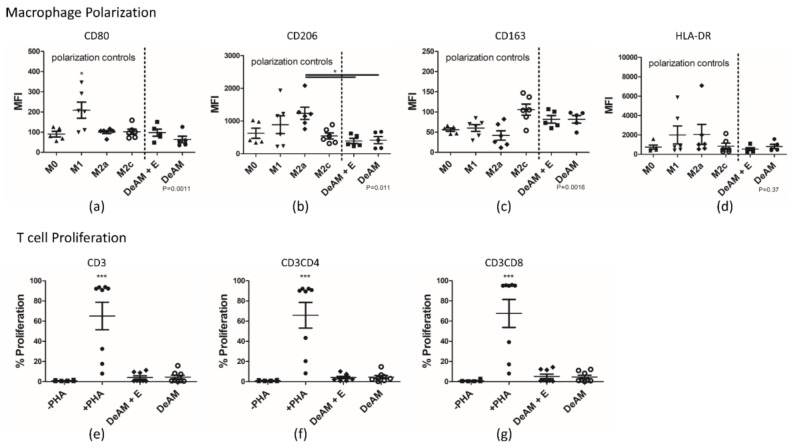
Polarization of M0 macrophages towards the pro-inflammatory M1 and anti-inflammatory M2a and M2c type was determined by flow cytometry after culturing for 48 h on DeAM + E and DeAM. Mean fluorescence intensities (MFI) for (**a**) CD80 (* *p* < 0.05 to all groups); (**b**) CD206; (**c**) CD163 and (**d**) HLA-DR marker expression are depicted. Polarization control groups (M0, M1, M2a, M2c) were cultured on standard plastic surface. PBMC were labeled with CFSE and cultured for 5 days on DeAM and DeAM + E. Proliferation was determined by flow cytometry for (**e**) CD3^+^; (**f**) CD3CD4^+^ and (**g**) CD3CD8^+^ T cells (*** *p* < 0.001 to all groups). Negative (−PHA) and positive stimulated (+PHA) controls were cultured on standard culture surface, *n* ≥ 5.

Finally, we examined the impact of hgECM coating on macrophage polarization towards the M1 or M2 type by examining the magnitude of surface marker expression. In general, ECM has the potential to induce M2 polarization [19] and might also influence T cell proliferation. Therefore, we tested the behavior of macrophages and T cells in contact with the patch materials. Immunophenotyping (CD surface antigens) and proliferation by Carboxyfluorescein Succinimidyl Ester (CFSE) staining was determined by flow cytometry (Figure 6).

After macrophage polarization, the M1 macrophage polarization control group represents the pro-inflammatory positive control and displayed upregulated expression of the CD80 marker (Figure 6a). The anti-inflammatory control groups M2a and M2c expressed predominantly CD206 (Figure 6b) and CD163 (Figure 6c) and on higher levels, respectively. Although expected to be highly expressed in M1 macrophages, HLA-DR (Figure 6d) did not significantly differ between control groups and amniotic scaffolds. Macrophages cultured on DeAM or DeAM + E showed a similar expression profile. In both M2 control groups as well as the M0 control group, no upregulation of CD80 was observed. Similar to the M2c control, macrophages on both surfaces showed the trend of higher expression of CD163 and no change in CD206 expression, suggesting that an M2c polarization took place.

As expected, PHA-stimulation of PBMCs induced a substantial increase in the proliferation of CD3^+^ T cells as well as with a comparable proportion in the CD4^+^ and CD8^+^ T cell subpopulations (Figure 6e–g). In contrast, culture on DeAM and DeAM + E scaffolds alone did not induce a significant proliferation of T cells.

## 3. Discussion

In the present work, we show that ECM isolated from human myocardium has the potential to modify the regenerative properties of a biological scaffold—decellularized human amniotic membrane—so that it is potentially better suited for therapeutic application in the diseased heart.

ECM has recently been shown to have great potential in regenerative and differentiation-guiding characteristics. Specific and crucial tissue characteristics are determined by the unique multi-protein composition of the ECM [20]. It is primarily produced by fibroblasts, and its unique biological properties can vary in response to physical changes. In the heart, ECM plays an essential role in myocardial infarction and other pathologies leading to heart failure [21]. Cardiac ECM has been isolated from different species, such as zebrafish [22], cow [23], rat [24] or pig [25], and is deemed a promising source of organ-specific, multi-protein preparations for intramyocardial or epicardial application. ECM also supports an anti-inflammatory response [26,27,28] and induces specific gene expression [29,30]. Processing of the naïve decellularized ECM to microparticles, followed by reconstitution to a hydrogel, widens the potential application range [31] and has been shown to be feasible [32] and translatable for cardiac tissue [33].

The use of animal tissue for experimental and clinical ECM applications is well established [10,23,25]. For example, cardiac ECM from Zebrafish was processed into microparticles to enable in vivo analyses by intramyocardial injection [22]. Porcine ECM from different organs, processed to a hydrogel, was shown to hold the potential to induce cardiac remodeling and increase cardiac function in large animal models [33]. However, the possibilities of applying human material have been less thoroughly researched. For instance, Godier-Furnémont et al. explored the possibility to design a patch with human ECM slices, but the addition of fibrin glue was necessary to stabilize the patch [12]. Moreover, the dimensions of an intact ECM slice were limited by the size of the available source myocardium. In contrast, our approach allows the use of human cardiac ECM to completely cover a sizable scaffold material, facilitating future clinical applications. We have previously shown that hcECM induces selectively cardiac differentiation/maturation and is cytoprotective for cardiomyocytes as well as stem and progenitor cells [13,14]. However, because it is a very fragile substance, it requires a mechanically stable scaffold material for application onto or into the heart. Therefore, ECM homogenization is a critical additional processing step that could open up new possibilities in regenerative medicine [34]. Additionally, we have already been able to show that processing the human cardiac ECM in this way does not impact its cytoprotective capacity [15].

Previous attempts to improve the biologic function of scaffold materials were largely made using standardized proteins such as gelatin [11] or fibrin [35]. More recently, Faulk et al. demonstrated the feasibility of covering polypropylene mesh scaffold with porcine dermal ECM [31]. However, using a polymer-based scaffold material in the heart risks inducing pro-inflammatory reactions, and therefore graft rejection, due to its chemical composition [8].

To avoid the potential complications accompanying the use of a synthetic scaffold material, we decided to use DeAM as an ECM scaffold which, in its naive form, we previously tested in vivo [6]. Therefore, the additional coating of hgECM does not dramatically change the characteristics of the resulting composite material, but, ideally, simply adds cardiac specificity. In line with this hypothesis, we did not observe a difference in the mechanical properties of coated and uncoated DeAM. While artificial polymer materials in general react linearly [5], the amniotic membrane can compensate a certain amount of stress by its natural flexibility, later followed by a linear elastic behavior until it reaches the maximum stress/strain slope. Material stiffness can also have a significant impact on cell behavior [36,37] but it is not affected by hgECM coating. Additionally, we were concerned about the biologic activity of the myocardial ECM product in terms of lineage support and cytoprotection which is most likely determined by its tissue-specific protein composition [14]. By mass spectrometry, we identified ECM proteins such as chitinase, a participant in pathogen defense and preventer of apoptosis by AKT activation, the Myosin binding protein C, involved in filament binding and muscle contractions, and the protein ecdysoneless homolog, which regulates p53 stability. Even though it remains unclear how ECM precisely exerts its cytoprotective effects, the presence of those partly intracellular proteins support the objective that coating of DeAM with hgECM increases cell proliferation and reduces cell necrosis as well as pro-inflammatory cytokine secretion. We observed that monocytes were not activated and secreted less IL-6 and TNF-α if cultured on coated scaffold in an activated state, suggesting the potential to ameliorate the inflammation present in infarcted myocardium.

In addition, according to our expectations both tested scaffolds did not induce a pro-inflammatory macrophage reaction (i.e., M1 polarization) but rather trend to induce a polarization towards the anti-inflammatory M2-type. The kind of material has a sensitive effect on the macrophage polarization behavior. It was shown that an electrospun polymer could induce a polarization towards the pro-inflammatory M1-type [8,27] whereas the introduction of collagen [38] or ECM [27] into the scaffold design reduced or even reversed that effect. Moreover, Ariganello et al. [39] highlights in particular decellularized pericardial tissue being supportive for guiding macrophage polarization towards anti-inflammatory tissue-remodeling state underlining the beneficial impact of additional hgECM coating.

We primarily aim at using the DeAM + E composite material for sustained delivery of biologics (cell products, reprogramming factors etc.) to the epicardial surface of the infarcted heart, since the epicardium is known to be highly involved in cardiac regeneration processes. In zebrafish and young mice, it has been shown that the epicardium can completely regenerate the infarcted myocardium [2,40,41]. To support cardiac regeneration via the epicardium, patches with cellular and protein-based [35,42] or polymeric [5,11,43] background have been designed by other groups. Epicardial cells were shown to have regenerative and angiogenic potential not only in animals but also when isolated from human hearts [44]. We are convinced that the limited regenerative capacity of the human heart can be augmented by tailored epicardial patch designs.

In summary, we developed a cell-free human amniotic membrane/myocardial ECM composite scaffold material with favorable in vitro biological and mechanical properties that has the potential be used in large animal experiments and ultimately clinical studies. As a cell-free material, the production process can readily be scaled-up under GMP conditions. Further in vivo testing will reveal whether the DeAM + E material alone is able to significantly modify post-infarct remodeling processes. In any event, it may serve as a universal platform for epicardial delivery of a broad spectrum of cells and therapeutic agents.

## 4. Material and Methods

### 4.1. Tissue Source

Left ventricular myocardium was collected from explanted hearts from patients who underwent heart transplantation for end-stage dilated cardiomyopathy. All patients were in New York Heart Association class II or IV, with a left ventricular ejection fraction <20%, and were not on mechanical circulatory support. Hepatitis B or C and HIV infection were ruled out preoperatively. The study protocol conforms to the ethical principles outlined in the Declaration of Helsinki. Patients provided informed consent for the use of the tissue for research purposes, and the process of tissue collection was approved by the Institutional Review Board and ethics committee of Charité—Universitätsmedizin Berlin (EA4/028/12, 20 June 2012).

Human full term placentas (*n* = 17) were anonymously obtained after cesarean delivery of healthy and uncomplicated pregnancies from women who had given written informed consent for use of the placenta for research purposes.

### 4.2. Human Amniotic Membrane Processing

Excess blood was removed by washing with Hank’s balanced salt solution (HBSS; Life Technologies, Carlsbad, CA, USA. The amniotic membrane (AM) was mechanically peeled from the underlying chorion and washed several times in HBSS to remove blood. Subsequently, AM was washed with phosphate buffered saline without calcium and magnesium (PBS^−/−^, Life Technologies) and placed in lysis buffer (10 mM Tris and 0.1% EDTA (both Carl-Roth, Karlsruhe, Germany) for 1 h at room temperature, followed by incubation in 0.5% sodium dodecyl sulfate (SDS; Carl-Roth) solution for 4 h at room temperature with constant stirring. Next, the AM was washed in PBS three times at room temperature and finally overnight at 4 °C under constant agitation.

Decellularized AM (DeAM) was cut into appropriate pieces and lyophilized.

### 4.3. ECM Processing

Human myocardium was harvested under sterile conditions and stored for no longer than 48 h at 4 °C before sectioning. Human Cardiac Extracellular Matrix (hcECM) isolation and processing was performed as described [45]. The myocardium was cut into cubes with an edge length of approximately 1 cm, embedded into Tissue Tec O.C.T (Sakura Inc., Alphen aan den Rijn, The Netherlands), sectioned in 300 µm thick slices using a CM 3050S cryostat (Leica, Wetzlar, Germany) , and stored at −80 °C before further processing. Subsequently, tissue slices were shaken for 2 h with lysis buffer (10 mM Tris, 0.1% *w*/*v* EDTA, pH 7.4; Carl-Roth) followed by SDS (0.5% *w*/*v* in PBS; Carl-Roth) incubation for 6 h under constant agitation at room temperature. Then, the slices were washed three times with PBS for 10 min to remove SDS, followed by an overnight washing period with Dulbecco’s phosphate buffered saline (DPBS) including 100 U/mL penicillin/streptomycin and nystatin to guarantee complete removal of SDS. Finally, the tissue was immersed in fetal bovine serum (FBS; Biochrom, Cambridge, UK) for 3 h at 37 °C followed by three washes with DPBS/penicillin/streptomycin/nystatin for 10 min each. Prior to further processing and/or experimentation, cECM slices were stored at 4 °C for up to 2 weeks.

To process decellularized matrix, slices were snap-frozen in liquid nitrogen, lyophilized and pulverized using the Precellys ceramic Kit 1.4 mm (PEQLAB Biotechnologie, Erlangen, Germany) and the Minilys homogenizer (PEQLAB Biotechnologie) at a rate of six runs per 1 min at 4000 rpm. Resulting particles were resolved in distilled water and filtered using a 200 µm transfusion filter system to remove larger particles, lyophilized again for 48 h, and stored at −80 °C. For homogenization, 1 mg/mL pepsin from porcine gastric mucosa (Sigma-Aldrich, Saint Louis, MO, USA) was dissolved in 0.01 M HCl, and incubated with 10 mg/mL hcECM particles for 48 h under constant agitation. To inhibit the Pepsin activity, the hcECM solution was neutralized on ice and brought to physiological pH by adding pre-cooled 10× PBS and 0.1 M NaOH. Finally, the concentration was set to 8 mg/mL by dilution using 1× PBS and hcECM Hydrogel (hgECM) was stored at 4 °C for not longer than 24 h before use.

### 4.4. Preparation of hgECM Coated Composite Materials

With an 8 mm Biopsy-Punch (PFM Medical, Cologne, Germany), the DeAM was cut into discs and lyophilized. One disc of DeAM was placed into each well of a 48-well plate and held in place by rings cut from silicone tubing (Ismatec, Wertheim, Germany) providing an inner diameter equal to 96-well format, and washed with PBS. The samples were completely coated by adding 150 µL/cm^2^ hgECM solution and dried for 48 h at 37 °C in a non-humidified incubator. After two gentle washes with PBS, the coated membranes were used for experiments. DeAM and DeAM coated with hgECM (DeAM + E) were tested.

### 4.5. Scanning Electron Microscopy

Samples were washed twice in PBS and fixed with 2.5% grade I glutaraldehyde (Sigma-Aldrich) for 30 min at room temperature. Subsequently, samples were washed again in PBS and dried using 5 min incubation steps in increasing Ethanol (Carl-Roth) concentration (mixed with ddH_2_O) (30%, 50%, 70%, 80%, 90%, 95%) followed by two final incubations in 100% Ethanol. Ethanol was removed by two incubations in Hexamethyldisilazane (Sigma-Aldrich) for 10 min each. Samples were air-dried overnight under a fume hood. Then, samples were placed on a stamp (Agar Scientific, Essex, UK) with 12 mm PLANO Tab (Plano GmbH, Wetzlar, Germany) and sputter coated with gold for 30 s (JFC-1200 Fine Coater; JEOL). Finally, samples were imaged using the JCM 6000 benchtop SEM (JEOL, Tokyo, Japan) in high vacuum mode at 10  kV.

### 4.6. Mechanical Testing of Amniotic Membrane Stress Measurement

Uniaxial pulling tests were performed in a wet state for the different groups under a BOSE testing bench (BOSE ElectroForce^®^ TestBench, TA Instruments, New Castle, DE, USA) using fixed clamps. Sample length was set to 1 cm, while thickness and width were individually measured using a micrometer (precision: ±0.001 µm) and a caliper (precision: ±0.01 mm) (Mitutoyo Corporation, Kawasaki, Japan), respectively. Crosshead speed was set to 0.05 mm/s and pulling up to 0.9 strain. Elastic moduli were calculated as the slope of the most linear region presented on the stress over strain curve. Stress and strain were obtained in agreement with the initial cross sectional area and length of each sample. Maximum stress was obtained as the maximum load before sample rupture.

### 4.7. Mass Spectrometry

The hgECM (140 µg) was resolved in 40 µL P-buffer (0.05 M Tris Base pH 7.5 (Sigma-Aldrich), 0.05 M potassium chloride (Merck, Darmstadt, Germany), 0.11 M Chaps (SERVA, Heidelberg, Germany), 20% glycerine (Merck), PhosSTOP (Roche, Basel, Switzerland), cOmplete (Roche)) and sonicated on ice. Then, 200 µL UA (0, 1 M Tris HCL pH 8.5 (Sigma-Aldrich) and 8 M Urea (Sigma-Aldrich)) were added and incubated for 10 min at room temperature. The solution was transferred into an amicon filter (10 kDa cut off, Merck), followed by buffer exchange to trypsin solution, (12 µg trypsin in 50 mM ammonium bicarbonate; Promega, Madison, WI, USA) and incubated at 37 °C overnight. Subsequently, ABC-buffer (50 mM ammonium bicarbonate; Sigma-Aldrich) was added and the solution centrifuged for 10 min at 14,000 rpm at room temperature. The flow-through was desalted using ZipTip C18 (Merck) pipet tips according the manufacturer’s protocol. Peptide samples were dried in a SpeedVac (Thermo-Fisher, Waltham, MA, USA) concentrator and extracted afterwards in 20 µL 0.1% trifluoroacetic acid (TFA) for 15 min at room temperature. Analysis was performed using UPLC (Dionex Ultimate 3000, Thermo-Fisher) ESI-QTOF-mass spectrometer (Imapct II, bruker daltonics, Billerica, MA, USA). Mass spectra were evaluated using MASCOT software (version number 2.2, Matrix Science, Boston, MA, USA) automatically searching the SwissProt 51.9 database (Human 553474 sequences; 198069095 residues, Cambridgeshire, UK). MS/MS ion search was performed with the following set of parameters: (i) taxonomy: *Homo sapiens* (human) (20175 sequences); (ii) proteolytic enzyme: trypsin; (iii) maximum of accepted missed cleavages: 1, (iv) mass value: monoisotopic, (v) peptide mass tolerance 10 ppm; (vi) fragment mass tolerance: 0.05 Da; and (vii) variable modifications: oxidation. No fixed modifications were considered. Only proteins with scores corresponding to *p* < 0.05 were considered. The cut-off score for individual peptides was equivalent to *p* < 0.05 for each peptide as calculated by MASCOT (version number 2.2).

### 4.8. Cell Culture

All cell types were negatively tested for mycoplasma contamination.

#### 4.8.1. HL-1

Murine HL-1 cardiomyocytes (HL-1), immortalized using the simian virus SV40 T-antigen under the control of an atrial natriuretic factor (ANF) promotor [46], were generously provided by William C. Claycomb (Louisiana State University, New Orleans, LA, USA) and used at passages 18–44. Cells were cultured in Claycomb medium with 10% HL-1 Cell Screened FBS (Merck), 100 U/mL penicillin, 100 µg/mL streptomycin, 2 mM L-Glutamine (Gibco, Waltham, MA, USA) and 100 µM norepinephrine, and cultured on 0.02% gelatin/5 µg/mL fibronectin-coated (Sigma-Aldrich) cell culture flasks at 37 °C and 5% CO_2_.

#### 4.8.2. Human Cardiac Fibroblasts

Human cardiac fibroblasts (hCF; generously donated by S. van Linthout, BCRT, Berlin) were cultured in DMEM (Gibco), 10% FBS (Biochrom) added and 100 U/mL penicillin, 100 µg/mL streptomycin (Gibco).

#### 4.8.3. Human Immune Cells

Human immune cells (monocytes, macrophages and peripheral blood mononuclear cells) were cultured in VLE RPMI 1640 Medium (Biochrom), 10% hAB Serum (Sigma-Aldrich) 100 U/mL penicillin, 100 µg/mL streptomycin and 2 mM L-Glutamine (Gibco).

Peripheral blood mononuclear cells (PMBCs) were isolated from Buffy Coat (bought from Deutsches Rotes Kreuz with approval by the Institutional Review Board and ethics committee of Charité—Universitätsmedizin Berlin EA1/372/16) using Biocoll (Biochrom) density gradient.

Monocytes were isolated from PMBCs using the CD14^+^ Magnetic Cell Separation kit (Miltenyi Biotec, Bergisch Gladbach, Germany).

Macrophages were differentiated from isolated monocytes by a 7 day incubation in culture medium with 50 ng/mL M-CSF (Miltenyi Biotec) and collected with a cell scraper.

#### 4.8.4. Epicardial Derived Cells

Adult human atrial samples (auricles) were obtained during cardiac surgery as redundant material anonymously collected as surgical waste under general informed consent. Epicardial Derived Cells (EPDC) were isolated from human heart auricles as described [44]. Briefly, the epicardial layer was peeled off with sterile tweezers and incubated for 30 min in Trypsin/EDTA at 37 °C. Cells were separated with a cell strainer and cultured in a 1:1 mixture of Dulbecco’s modified Eagle’s medium (DMEM-glucose low; Invitrogen, Carlsbad, CA, USA) and Medium 199 (M199; Invitrogen) supplemented with 10% heat-inactivated FBS (Biochrom), 100 U/mL penicillin/streptomycin (Gibco) and 5 ng/mL TGFβ (EUROFINS) on 0.1% gelatin coated cell culture dish.

### 4.9. Simulated Ischemia

Simulated ischemia cells were cultured on patch material or cell culture dishes for 24 h before being exposed to glucose/serum deprivation (glucose- and FBS-free DMEM (Thermo-Fisher)), with 100 U/mL penicillin/streptomycin (Gibco), 1% O_2_/5% CO_2_) for 5 h in a Binder CB150 incubator (CB150, Binder, Tuttlingen, Germany).

### 4.10. Cell Behavior

#### 4.10.1. Cardiac Cell Adhesion

Cells were labelled with 5 µM Calcein (MoBiTec, Herrljunga, Sweden) following the manufacturer’s instructions. For determining adhesion rates, 125,000 HL-1 cells, or 20,000 hCF or EPDCs per cm^2^ were used. Cells were placed onto the scaffolds and incubated at 37 °C for 30 min, 60 min, or 120 min. Supernatants containing unbound cells were collected and cells were lysed with a final concentration of 1 °C% Triton-X100. Fluorescence intensity, representing the number of cells, was analyzed using the MithrasLB940 plate reader (Berthold Technologies, Bad Wildbad, Germany. Adherent cell number was calculated via comparison to a defined cell number standard, and the amount of adherent cells was determined.

#### 4.10.2. Necrosis (LDH)

Determination of cell necrosis was performed by measuring the release of Lactate Dehydrogenase (LDH) using the CytoTox-ONE™ Homogeneous Membrane Integrity Assay (Promega). Then, 125,000 HL-1 cells, or 20,000 hCF, or EPDCs per cm^2^, were cultured onto the scaffolds or on standard cell culture conditions for controls. Cells were either cultured on standard culture conditions for 24 h or additionally exposed to simulated ischemia. Next, supernatants were analyzed for LDH release according to manufacturer’s instructions, and fluorescence was measured using the MithrasLB940 plate reader (Berthold Technologies). Results were normalized to normal cell culture conditions.

#### 4.10.3. Cell Growth

Cell growth was analyzed using the BrdU Cell Proliferation ELISA Kit (Roche). To determine cell growth, 62,500 HL-1 cells, or 20,000 hCFs or EPDCs per cm² were cultured onto the scaffolds or standard cell culture conditions as controls. Cells were cultured under standard conditions for 24 h at 37 °C in a humidified incubator. Subsequently, labelling reagent was added and incubated for 5 h at 37 °C under normoxia or simulated ischemia. Afterwards, cells were analyzed for BrdU-incorporation according to protocol. Finally, the reaction was stopped by adding 1 M H_2_SO_4_, and 100 µm of the solution was transferred to a fresh 96-well plate. Read out was performed using the SpectraMax 340PC384 (Molecular Devices, San Jose, CA, USA).

### 4.11. Immunological Analyses

#### 4.11.1. Monocytes–Cytokine Secretion

To determine cytokine secretion, 100,000 monocytes were cultured onto patch materials and incubated for 24 h at 37 °C. For the positive control, medium was supplemented with 200 ng/mL Lipopolysaccharide (LPS). Supernatants were collected and cytokine secretion was detected using Human ELISA MAX™ Deluxe Kits (Thermo-Fisher) for IL-6, IL-10 and TNF-α following the manufacturer’s protocol.

#### 4.11.2. Staining Procedure for Flow Cytometry

Cells were collected and washed once in buffer (PBS, 1% FBS (Biochrom), 0.5% Sodium azide (Carl-Roth)). The supernatant was discarded completely and 50 µL staining solution in buffer containing all labeling antibodies was added and incubated for 30 min at 4 °C. Subsequently, the suspension was washed once with buffer and suspended in buffer containing 1% Paraformaldehyde (PFA, Carl-Roth) and analyzed the next day. The following antibodies were used for macrophage staining: CD163-Fitc (1:20, BioLegend, San Diego, CA, USA), CD80-PE (1:20, BioLegend), CD16-PerCPCy5.5 (1:200, BioLegend), CD206-APC (1:100, BioLegend), CD14-APCCy7 (1:100, BD, Franklin Lakes, NJ, USA), HLA-DR-PeCy7 (1:400, BioLegend), Life/Dead-V510 (1:100, Thermo-Fisher). Following antibodies were used for T cell staining in the proliferation setup: CD8-PE (1:50, Miltenyi Biotec), CD4-APC (1:100, BioLegend), CD3-APCCy7 (1:100, BioLegend).

#### 4.11.3. Peripheral Blood Mononuclear Cell Cytokine Secretion and T Cell Proliferation

CFSE stain for T cell proliferation was performed using the CFSE labeling procedure from the Total Cytotoxicity & Apoptosis Detection Kit (Biomol, Hamburg, Germany). Briefly, PBMCs (provided by Karen Bieback; Heidelberg) were adjusted to 1 × 10^7^ cells in 1 mL assay buffer, washed twice in assay buffer and a labeled 200 µL CFSE working solution was added to 1.8 mL cell suspension in assay buffer. The labeled suspension was incubated for 15 min at room temperature. The reaction was stopped by adding medium followed by two washing steps with assay buffer. Finally, cells were suspended in the medium and used for the experiment. Then, 150,000 PBMCs were cultured on patch materials for 5 days. The positive control medium was supplemented with 5 µg/mL Phytohaemagglutinin (PHA). Subsequently, cells were harvested and analyzed for proliferation of CD3^+^, CD3CD4^+^ and CD3CD8^+^ T cell proliferation by flow cytometry (gating strategy is provided in supplementary material Figure S2). Supernatants were collected and cytokine secretion was detected using Human ELISA MAX™ Deluxe Kits (Thermo-Fisher) for IL-6, IL-10 and TNF-α following the manufacturer’s protocol.

#### 4.11.4. Macrophage Polarization

To investigate macrophage polarization, 100,000 macrophages were cultured for 48 h on patch materials or cell culture dishes. To induce polarization towards M1, the culture medium was supplemented with 20 ng/mL INFy +100 ng/mL LPS. For M2a polarization, 20 ng/mL IL-4 and for M2c 20 ng/mL IL-10 was used.

After 2 days of culture, supernatants were collected for detection of cytokine secretion via ELISA using Human ELISA MAX™ Deluxe Kits (Thermo-Fisher) for IL-6, IL-10 and TNF-α following the manufacturer’s protocol. Cells were harvested using Accutase (Innovative Cell Technologies, San Diego, CA, USA)) and analyzed for polarization marker expression by flow cytometry (gating strategy is provided in supplementary material Figure S1).

### 4.12. Statistics

Data are shown as mean ± SEM. Comparisons passed normality and equal variance testing before the significance was tested. Differences between more than two groups were determined by one-way ANOVA with Bonferroni *t*-test for multiple comparisons. For two-group comparisons, a two-tailed student’s *t*-test was performed if the normality test was passed. The Mann-Whitney test was performed if non-normality was detected. Changes over time were tested by two-way ANOVA with Bonferroni’s correction. GraphPad Prism v. 5.03 (GraphPad, La Jolla, CA, USA) was used for data analysis and plotting. A *p*-value of *p* < 0.05 was considered significant.

## 5. Conclusions

The source and composition of applied extracellular matrix is crucial for specific application. Here, we demonstrated the additional coating of cell-free amniotic membrane scaffolds with processed human cardiac extracellular matrix. The novel resulting patch material not only specifically supports the culture and interaction of cardiac cells such as cardiomyocytes, epicardial cells, and cardiac fibroblasts, but also exerts immunomodulatory effects on cell subsets relevant for cardiac regeneration and tissue engineering. The newly designed patch system may therefore enhance the therapeutic capacity of epicardial composite materials and pave the way for improved cardiac regeneration attempts.

## Supplementary Materials

Supplementary materials can be found at http://www.mdpi.com/1422-0067/19/4/1032/s1.

## Abbreviations

ECM	extracellular matrix
hcECM	human cardiac extracellular matrix
hgECM	human cardiac extracellular matrix hydrogel
AM	amniotic membrane
DeAM	decellularized amniotic membrane
DeAM + E	decellularized amniotic membrane coated with human cardiac extracellular matrix hydrogel
PBMCs	peripheral blood mononuclear cells
