# Supplementary material for: Towards a Novel Patch Material for Cardiac Applications: Tissue-Specific Extracellular Matrix Introduces Essential Key Features to Decellularized Amniotic Membrane
Source: Int J Mol Sci. 2018 Mar 29;19(4):1032. doi: 10.3390/ijms19041032 (PMC5979550; doi:10.3390/ijms19041032)
Supplement: Supplementary file 1 [file ijms-19-01032-s001.pdf]

Supplementary Materials: Towards a Novel Patch Material for Cardiac Applications: Tissue-Specific Extracellular Matrix Introduces Essential Key Features to Decellularized Amniotic Membrane

Matthias Becker, Janita A. Maring, Maria Schneider, Aarón X. Herrera Martin, Martina Seifert, Oliver Klein, Thorsten Braun, Volkmar Falk and Christof Stamm

Table S1. Proteins in hgECM identified by mass spectrometry.

Accession	Name	Scores A	Scores B	Scores C	Matches
A2MG_HUMAN	Alpha-2-macroglobulin OS=Homo sapiens GN=A2M PE=1 SV=3		114.4	13.7	1
AATM_HUMAN	Aspartate aminotransferase, mitochondrial OS=Homo sapiens GN=GOT2 PE=1 SV=3	42.1		13.2	1
ACADV_HUMAN	Very long-chain specific acyl-CoA dehydrogenase, mitochondrial OS=Homo sapiens GN=ACADVL PE=1 SV=1	22.3		31.4	2
ACON_HUMAN	Aconitate hydratase, mitochondrial OS=Homo sapiens GN=ACO2 PE=1 SV=2	156.9		66.7	3
ACTA_HUMAN	Actin, aortic smooth muscle OS=Homo sapiens GN=ACTA2 PE=1 SV=1	1247.8	34.6	1149.1	8
ACTN2_HUMAN	Alpha-actinin-2 OS=Homo sapiens GN=ACTN2 PE=1 SV=1	806.3		835.8	9
ACTS_HUMAN	Actin, alpha skeletal muscle OS=Homo sapiens GN=ACTA1 PE=1 SV=1	840.3		990.5	7
ADT1_HUMAN	ADP/ATP translocase 1 OS=Homo sapiens GN=SLC25A4 PE=1 SV=4	360.9		401.3	7
ADT3_HUMAN	ADP/ATP translocase 3 OS=Homo sapiens GN=SLC25A6 PE=1 SV=4			231.7	5
ALBU_HUMAN	Serum albumin OS=Homo sapiens GN=ALB PE=1 SV=2	269.8	292.1	47.1	4
APOA1_HUMAN	Apolipoprotein A-I OS=Homo sapiens GN=APOA1 PE=1 SV=1	67.3	76.9	27.0	1

ARHL1_HUMAN	[Protein ADP-ribosylarginine] hydrolase-like protein 1 OS=Homo sapiens GN=ADPRHL1 PE=2 SV=1	48.3		31.0	1
AT2A1_HUMAN	Sarcoplasmic/endoplasmic reticulum calcium ATPase 1 OS=Homo sapiens GN=ATP2A1 PE=1 SV=1			151.1	1
ATP5I_HUMAN	ATP synthase subunit e, mitochondrial OS=Homo sapiens GN=ATP5I PE=1 SV=2			42.1	1
ATP6_HUMAN	ATP synthase subunit a OS=Homo sapiens GN=MT-ATP6 PE=1 SV=1	112.5		82.2	1
ATPA_HUMAN	ATP synthase subunit alpha, mitochondrial OS=Homo sapiens GN=ATP5A1 PE=1 SV=1	165.1		51.6	4
ATPB_HUMAN	ATP synthase subunit beta, mitochondrial OS=Homo sapiens GN=ATP5B PE=1 SV=3	108.1		155.2	1
ATPK_HUMAN	ATP synthase subunit f, mitochondrial OS=Homo sapiens GN=ATP5J2 PE=1 SV=3	308.0	29.7	580.1	2
CAV1_HUMAN	Caveolin-1 OS=Homo sapiens GN=CAV1 PE=1 SV=4	108.6	84.5	31.0	1
CHIA_HUMAN	Acidic mammalian chitinase OS=Homo sapiens GN=CHIA PE=1 SV=1			245.1	1
CMC1_HUMAN	Calcium-binding mitochondrial carrier protein Aralar1 OS=Homo sapiens GN=SLC25A12 PE=1 SV=2	73.7		30.9	1
CO1A1_HUMAN	Collagen alpha-1(I) chain OS=Homo sapiens GN=COL1A1 PE=1 SV=5	327.8	695.0	598.4	6
CO3_HUMAN	Complement C3 OS=Homo sapiens GN=C3 PE=1 SV=2	95.0	430.9	141.9	2
CO3A1_HUMAN	Collagen alpha-1(III) chain OS=Homo sapiens GN=COL3A1 PE=1 SV=4	18.5	299.4	614.5	2
CO4A2_HUMAN	Collagen alpha-2(IV) chain OS=Homo sapiens GN=COL4A2 PE=1 SV=4	294.3	428.4	642.1	5
CO5A3_HUMAN	Collagen alpha-3(V) chain OS=Homo sapiens GN=COL5A3 PE=1 SV=3		60.5	22.8	1
CO6A3_HUMAN	Collagen alpha-3(VI) chain OS=Homo sapiens GN=COL6A3 PE=1 SV=5	988.4	12200.9	27.0	1
COX1_HUMAN	Cytochrome c oxidase subunit 1 OS=Homo sapiens GN=MT-CO1 PE=1 SV=1	212.6		434.9	1

COX2_HUMAN	Cytochrome c oxidase subunit 2 OS=Homo sapiens GN=MT-CO2 PE=1 SV=1	15.4		40.8	2
COX3_HUMAN	Cytochrome c oxidase subunit 3 OS=Homo sapiens GN=MT-CO3 PE=1 SV=2	70.4		13.7	1
CRYAB_HUMAN	Alpha-crystallin B chain OS=Homo sapiens GN=CRYAB PE=1 SV=2	78.4	22.5	54.9	3
CTRO_HUMAN	Citron Rho-interacting kinase OS=Homo sapiens GN=CIT PE=1 SV=2			13.7	1
CX7A1_HUMAN	Cytochrome c oxidase subunit 7A1, mitochondrial OS=Homo sapiens GN=COX7A1 PE=1 SV=2	61.4		51.8	2
CYB_HUMAN	Cytochrome b OS=Homo sapiens GN=MT-CYB PE=1 SV=2	45.5		144.0	1
CYC_HUMAN	Cytochrome c OS=Homo sapiens GN=CYCS PE=1 SV=2	32.5		15.1	1
DDX6_HUMAN	Probable ATP-dependent RNA helicase DDX6 OS=Homo sapiens GN=DDX6 PE=1 SV=2	22.2	52.0	31.0	1
DERM_HUMAN	Dermatopontin OS=Homo sapiens GN=DPT PE=1 SV=2	85.1	84.1	27.0	1
DESM_HUMAN	Desmin OS=Homo sapiens GN=DES PE=1 SV=3	174.1		438.3	2
ECD_HUMAN	Protein ecdysoneless homolog OS=Homo sapiens GN=ECD PE=1 SV=1			22.8	1
EF1A2_HUMAN	Elongation factor 1-alpha 2 OS=Homo sapiens GN=EEF1A2 PE=1 SV=1	284.5		66.5	2
FABPH_HUMAN	Fatty acid-binding protein, heart OS=Homo sapiens GN=FABP3 PE=1 SV=4	141.1		31.0	1
FBN1_HUMAN	Fibrillin-1 OS=Homo sapiens GN=FBN1 PE=1 SV=3	248.7	107.6	41.4	3
FHL1_HUMAN	Four and a half LIM domains protein 1 OS=Homo sapiens GN=FHL1 PE=1 SV=4	102.9		94.3	1
FHL2_HUMAN	Four and a half LIM domains protein 2 OS=Homo sapiens GN=FHL2 PE=1 SV=3	42.9		46.3	1
FINC_HUMAN	Fibronectin OS=Homo sapiens GN=FN1 PE=1 SV=4	1698.4	9406.0	27.0	1

FLNC_HUMAN	Filamin-C OS=Homo sapiens GN=FLNC PE=1 SV=3	692.1		43.1	2
G3P_HUMAN	Glyceraldehyde-3-phosphate dehydrogenase OS=Homo sapiens GN=GAPDH PE=1 SV=3	204.4	125.2	105.8	3
GELS_HUMAN	Gelsolin OS=Homo sapiens GN=GSN PE=1 SV=1	66.9	41.2	13.7	1
GHITM_HUMAN	Growth hormone-inducible transmembrane protein OS=Homo sapiens GN=GHITM PE=1 SV=2	22.5		13.7	1
GPAA1_HUMAN	Glycosylphosphatidylinositol anchor attachment 1 protein OS=Homo sapiens GN=GPAA1 PE=1 SV=3			13.7	1
GYS1_HUMAN	Glycogen [starch] synthase, muscle OS=Homo sapiens GN=GYS1 PE=1 SV=2	47.7		38.3	1
H4_HUMAN	Histone H4 OS=Homo sapiens GN=HIST1H4A PE=1 SV=2	250.7	277.8	23.0	2
HBB_HUMAN	Hemoglobin subunit beta OS=Homo sapiens GN=HBB PE=1 SV=2	394.3	1418.0	28.3	1
HEMO_HUMAN	Hemopexin OS=Homo sapiens GN=HPX PE=1 SV=2			27.0	1
HHATL_HUMAN	Protein-cysteine N-palmitoyltransferase HHAT-like protein OS=Homo sapiens GN=HHATL PE=2 SV=1	227.9		23.5	2
HS71A_HUMAN	Heat shock 70 kDa protein 1A OS=Homo sapiens GN=HSPA1A PE=1 SV=1	37.7	145.9	89.1	2
HSPB1_HUMAN	Heat shock protein beta-1 OS=Homo sapiens GN=HSPB1 PE=1 SV=2	39.7	142.8	152.9	1
IDH3G_HUMAN	Isocitrate dehydrogenase [NAD] subunit gamma, mitochondrial OS=Homo sapiens GN=IDH3G PE=1 SV=1			27.0	1
IGHG1_HUMAN	Ig gamma-1 chain C region OS=Homo sapiens GN=IGHG1 PE=1 SV=1	26.9	204.3	88.2	1
IGKC_HUMAN	Ig kappa chain C region OS=Homo sapiens GN=IGKC PE=1 SV=1	96.0		60.1	2
K2C1_HUMAN	Keratin, type II cytoskeletal 1 OS=Homo sapiens GN=KRT1 PE=1 SV=6		95.5	16.9	2

KCRS_HUMAN	Creatine kinase S-type, mitochondrial OS=Homo sapiens GN=CKMT2 PE=1 SV=2	23.5		13.3	1
KPYM_HUMAN	Pyruvate kinase PKM OS=Homo sapiens GN=PKM PE=1 SV=4	27.5	116.4	27.0	1
LAMA2_HUMAN	Laminin subunit alpha-2 OS=Homo sapiens GN=LAMA2 PE=1 SV=4	45.5	68.3	26.3	2
LDB3_HUMAN	LIM domain-binding protein 3 OS=Homo sapiens GN=LDB3 PE=1 SV=2	103.2		90.5	4
LDHB_HUMAN	L-lactate dehydrogenase B chain OS=Homo sapiens GN=LDHB PE=1 SV=2			95.7	1
LMNA_HUMAN	Prelamin-A/C OS=Homo sapiens GN=LMNA PE=1 SV=1	268.5	1000.4	27.0	1
LRC47_HUMAN	Leucine-rich repeat-containing protein 47 OS=Homo sapiens GN=LRRC47 PE=1 SV=1			26.9	1
M2OM_HUMAN	Mitochondrial 2-oxoglutarate/malate carrier protein OS=Homo sapiens GN=SLC25A11 PE=1 SV=3	36.1		49.8	2
MFAP5_HUMAN	Microfibrillar-associated protein 5 OS=Homo sapiens GN=MFAP5 PE=1 SV=1	76.6	413.9	65.8	2
MGST3_HUMAN	Microsomal glutathione S-transferase 3 OS=Homo sapiens GN=MGST3 PE=1 SV=1	662.2		941.8	2
MIC60_HUMAN	MICOS complex subunit MIC60 OS=Homo sapiens GN=IMMT PE=1 SV=1			13.3	1
MPC2_HUMAN	Mitochondrial pyruvate carrier 2 OS=Homo sapiens GN=MPC2 PE=1 SV=1	34.0		22.6	1
MPCP_HUMAN	Phosphate carrier protein, mitochondrial OS=Homo sapiens GN=SLC25A3 PE=1 SV=2	234.2	29.1	89.6	2
MTCH2_HUMAN	Mitochondrial carrier homolog 2 OS=Homo sapiens GN=MTCH2 PE=1 SV=1	372.2		19.8	1
MYH7_HUMAN	Myosin-7 OS=Homo sapiens GN=MYH7 PE=1 SV=5	3052.7		2949.5	22
MYL3_HUMAN	Myosin light chain 3 OS=Homo sapiens GN=MYL3 PE=1 SV=3	26.6		27.0	1

MYOM1_HUMAN	Myomesin-1 OS=Homo sapiens GN=MYOM1 PE=1 SV=2	142.0		38.5	3
MYOM2_HUMAN	Myomesin-2 OS=Homo sapiens GN=MYOM2 PE=1 SV=2	113.8		90.2	2
MYOM3_HUMAN	Myomesin-3 OS=Homo sapiens GN=MYOM3 PE=2 SV=1	137.1		197.3	4
MYOZ2_HUMAN	Myozenin-2 OS=Homo sapiens GN=MYOZ2 PE=1 SV=1	57.8		31.0	1
MYPC3_HUMAN	Myosin-binding protein C, cardiac-type OS=Homo sapiens GN=MYBPC3 PE=1 SV=4	880.9		1077.8	13
NDUAB_HUMAN	NADH dehydrogenase [ubiquinone] 1 alpha subcomplex subunit 11 OS=Homo sapiens GN=NDUFA11 PE=1 SV=3	73.9		27.0	1
NDUB5_HUMAN	NADH dehydrogenase [ubiquinone] 1 beta subcomplex subunit 5, mitochondrial OS=Homo sapiens GN=NDUFB5 PE=1 SV=1			27.0	1
NNTM_HUMAN	NAD(P) transhydrogenase, mitochondrial OS=Homo sapiens GN=NNT PE=1 SV=3	126.3		152.9	1
NU4M_HUMAN	NADH-ubiquinone oxidoreductase chain 4 OS=Homo sapiens GN=MT-ND4 PE=1 SV=1	272.0		43.0	2
NU5M_HUMAN	NADH-ubiquinone oxidoreductase chain 5 OS=Homo sapiens GN=MT-ND5 PE=1 SV=2	390.1		185.9	3
OBSCN_HUMAN	Obscurin OS=Homo sapiens GN=OBSCN PE=1 SV=3	132.1		361.5	5
PDLI5_HUMAN	PDZ and LIM domain protein 5 OS=Homo sapiens GN=PDLIM5 PE=1 SV=5	61.0	22.9	44.5	2
PGBM_HUMAN	Basement membrane-specific heparan sulfate proteoglycan core protein OS=Homo sapiens GN=HSPG2 PE=1 SV=4	433.9	2232.2	22.8	1
PRDX6_HUMAN	Peroxiredoxin-6 OS=Homo sapiens GN=PRDX6 PE=1 SV=3		70.2	19.3	1
PROS_HUMAN	Vitamin K-dependent protein S OS=Homo sapiens GN=PROS1 PE=1 SV=1			27.0	1

QCR10_HUMAN	Cytochrome b-c1 complex subunit 10 OS=Homo sapiens GN=UQCR11 PE=3 SV=1			19.4	1
RL13A_HUMAN	60S ribosomal protein L13a OS=Homo sapiens GN=RPL13A PE=1 SV=2	20.8	85.6	25.2	1
RYR2_HUMAN	Ryanodine receptor 2 OS=Homo sapiens GN=RYR2 PE=1 SV=3	163.0		252.9	2
SC24C_HUMAN	Protein transport protein Sec24C OS=Homo sapiens GN=SEC24C PE=1 SV=3		40.3	13.7	1
SCO2_HUMAN	Protein SCO2 homolog, mitochondrial OS=Homo sapiens GN=SCO2 PE=1 SV=3			13.7	1
SMYD1_HUMAN	Histone-lysine N-methyltransferase SMYD1 OS=Homo sapiens GN=SMYD1 PE=1 SV=1	159.0		184.1	1
SRBS1_HUMAN	Sorbin and SH3 domain-containing protein 1 OS=Homo sapiens GN=SORBS1 PE=1 SV=3	280.1		149.3	1
TANC1_HUMAN	Protein TANC1 OS=Homo sapiens GN=TANC1 PE=1 SV=3			13.6	1
TBA1A_HUMAN	Tubulin alpha-1A chain OS=Homo sapiens GN=TUBA1A PE=1 SV=1		394.5	58.4	2
TBB2A_HUMAN	Tubulin beta-2A chain OS=Homo sapiens GN=TUBB2A PE=1 SV=1			79.3	1
TGM2_HUMAN	Protein-glutamine gamma-glutamyltransferase 2 OS=Homo sapiens GN=TGM2 PE=1 SV=2	464.6	582.2	31.7	2
THIM_HUMAN	3-ketoacyl-CoA thiolase, mitochondrial OS=Homo sapiens GN=ACAA2 PE=1 SV=2			20.1	1
TIMP3_HUMAN	Metalloproteinase inhibitor 3 OS=Homo sapiens GN=TIMP3 PE=1 SV=2	84.6	21.1	140.0	2
TINAL_HUMAN	Tubulointerstitial nephritis antigen-like OS=Homo sapiens GN=TINAGL1 PE=1 SV=1	224.5		60.9	3
TITIN_HUMAN	Titin OS=Homo sapiens GN=TTN PE=1 SV=4	31133.7		25097.0	294
TMCO4_HUMAN	Transmembrane and coiled-coil domain-containing protein 4			152.9	1

	OS=Homo sapiens GN=TMCO4 PE=2 SV=1				
TPM1_HUMAN	Tropomyosin alpha-1 chain OS=Homo sapiens GN=TPM1 PE=1 SV=2	88.2	39.3	58.5	3
TSP1_HUMAN	Thrombospondin-1 OS=Homo sapiens GN=THBS1 PE=1 SV=2	268.3	441.7	23.5	2
VDAC1_HUMAN	Voltage-dependent anion-selective channel protein 1 OS=Homo sapiens GN=VDAC1 PE=1 SV=2	158.1		75.7	2
VDAC2_HUMAN	Voltage-dependent anion-selective channel protein 2 OS=Homo sapiens GN=VDAC2 PE=1 SV=2	176.1		13.7	1
VDAC3_HUMAN	Voltage-dependent anion-selective channel protein 3 OS=Homo sapiens GN=VDAC3 PE=1 SV=1	85.7		24.2	2
VTNC_HUMAN	Vitronectin OS=Homo sapiens GN=VTN PE=1 SV=1	139.0	202.1	40.9	1
WDR1_HUMAN	WD repeat-containing protein 1 OS=Homo sapiens GN=WDR1 PE=1 SV=4			27.0	1

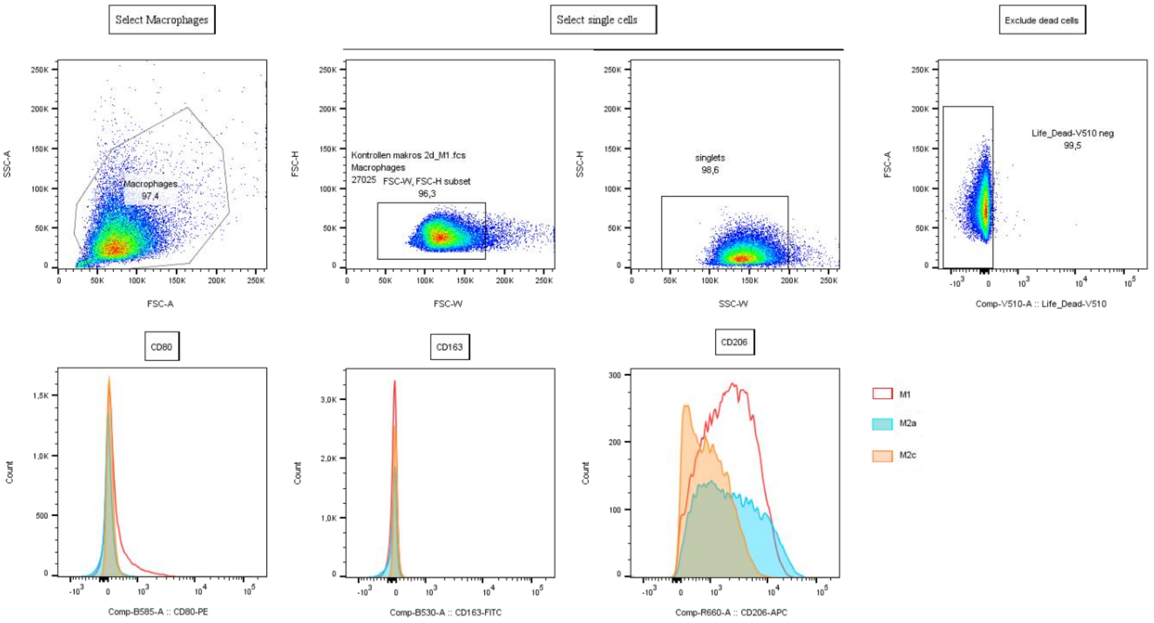

Figure S1. Representative flow cytometry gating strategy for macrophage polarization.

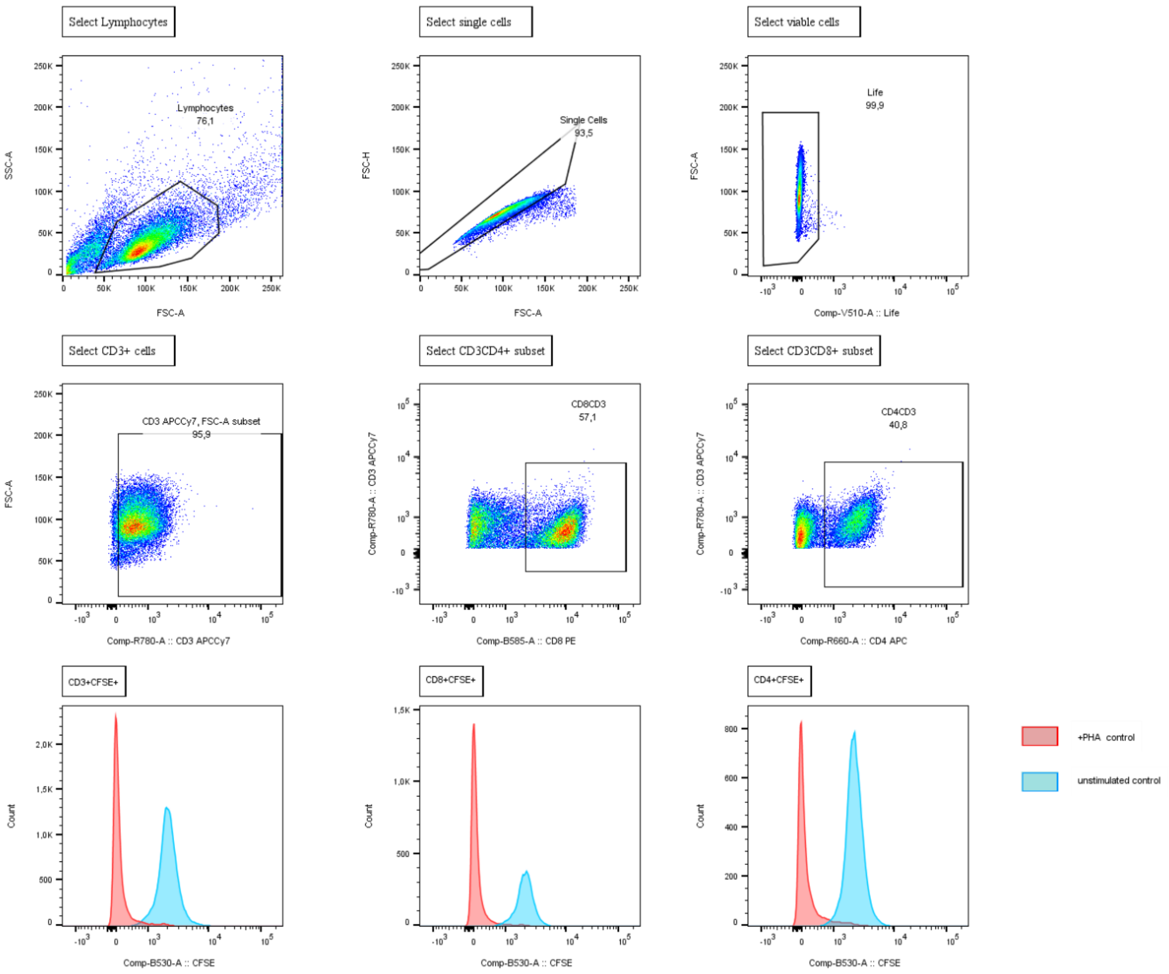

Figure S2. Representative flow cytometry gating strategy for T cell proliferation.
